# Spontaneous Reduction in the Intermetatarsal Angle in Distal First Metatarsal Osteotomies with No Lateral Head Displacement in Hallux Valgus

**DOI:** 10.3390/biomedicines12071438

**Published:** 2024-06-27

**Authors:** Jean-Yves Coillard, Romain Rey, Alessandro Civinini, Fabien Billuart, Eli Schmidt, Cesar de Cesar Netto, Riccardo Sacco, Matthieu Lalevée

**Affiliations:** 1 Clinique du Parc, Elsan Group, 155 Boulevard de Stalingrad, 69006 Lyon, France; jy.coillard@gmail.com; 2 Orthopedic and Trauma, Surgery Department, Rouen University Hospital, 37 Boulevard Gambetta, 76000 Rouen, France; romain.rey.16@gmail.com (R.R.); riccardosacco.ortho@gmail.com (R.S.); 3 Orthopedic Unit, Department of Health Sciences, University of Florence, C.T.O. Largo Palagi 1, 50139 Firenze, Italy; alessandro.civinini@unifi.it; 4 Laboratoire d’Analyse du Mouvement, Institut de Formation en Masso-Kinésithérapie Saint Michel, 75015 Paris, France; fbilluart@gmail.com; 5 ERPHAN Research Unit, UR 20201, University of Versailles Saint Quentin, 78000 Versailles, France; 6 Department of Orthopedics and Rehabilitation, University of Iowa, Iowa City, IA 52242, USA; eli-schmidt@uiowa.edu; 7 Department of Orthopedic Surgery, Duke University, Durham, NC 27710, USA; cesar.netto@duke.edu; 8 CETAPS UR 3832, Research Center for Sports and Athletic Activities Transformations, University of Rouen Normandy, 76821 Mont-Saint-Aignan, France

**Keywords:** hallux valgus, pronation, supination, distal metatarsal articular angle, intermetatarsal angle

## Abstract

Background: The outcomes of first metatarsal (M1) distal osteotomies in hallux valgus (HV) can be improved, especially for intermetatarsal angle (IMA) correction, which is mainly based on lateral displacement of the M1 head (i.e., translation) through the osteotomy. Conversely, there is a spontaneous reduction in the IMA in first metatarsophalangeal joint (MTP1) arthrodesis. But we do not know whether this can be applied to distal osteotomies. We propose a distal osteotomy, called 3D chevron, which combines supination and varization of the M1 head. This might realign soft tissues around the MTP1, potentially leading to a spontaneous reduction in the IMA by an analogous mechanism to MTP1 fusion. Therefore, our study aimed to assess whether spontaneous reductions in IMAs exist in distal M1 osteotomies in the absence of lateral translations of M1 heads. Methods: A prospective continuous series of 25 3D chevrons was performed. Two groups were formed during surgery. Patients requiring no M1 head lateral displacement were included in the “successful correction without translation” group, and patients requiring M1 head lateral displacement were included in the “failed correction without translation” group. Radiographic analysis was performed preoperatively and at 1 year postoperatively. Results: Twenty-two women and three men, with a mean age of 44.8 ± 14.2 years and a mean body mass index of 22.6 ± 4.1 kg/m^2^, underwent follow-up at one year after surgery. The “successful correction without translation” group was composed of HV with milder deformities (13/25 HVs, median preoperative IMA = 13 (IQR 2)) compared to the “failed correction without translation” group (median IMA = 16 (IQR 2.25) *p* < 0.001). Spontaneous reductions in IMAs were observed in the “successful correction without translation” group, with a median decrease in the IMA of 6 degrees (CI95%[5.5; 8.0]; *p* < 0.001) between preoperative and 1-year radiographs. Conclusion: Distal osteotomies allow for spontaneous reduction in the IMA in HV. First metatarsal head translation through an osteotomy should not be considered as the only procedure to correct IMAs in distal osteotomies.

## 1. Introduction

Hallux valgus (HV) is a common and potentially disabling deformity of the foot’s first ray, which often requires surgery if medical treatments fail. One of the most common surgical options is distal first metatarsal (M1) osteotomies. The outcomes of these osteotomies can be improved [1,2], with many recurrences of the deformity observed in the long term [3,4,5]. Furthermore, distal osteotomies are less effective than basal osteotomies in correcting the intermetatarsal angle (IMA) [6]. The correction of this angle in distal osteotomies is primarily based on the lateral translation of the head of the M1 through the osteotomy. The width of the M1 head can limit this movement, with extreme translation resulting in minimal bone contact and a decrease in the stability of the osteotomy. Some authors suggest translating the head beyond the width of M1 and then compensating for the lack of stability by increasing the quantity of osteosynthesis, as it is commonly done in minimally invasive chevron and Akin osteotomies (MICAs) for instance [7,8]. In this study, we propose a counterintuitive point of view. It has been observed that the IMA spontaneously reduces following arthrodesis of the first metatarsophalangeal joint (MTP1) [9,10]. This phenomenon, the mechanism of which has not yet been elucidated, has not yet been studied in conservative osteotomies. In MTP1 arthrodesis, it is suspected that the realignment of soft tissues around MTP1 is the root cause of this spontaneous reduction in the IMA [9,10]. We assumed that correction through conservative osteotomies of the bone deformities present in HV could also facilitate the realignment of the surrounding musculotendinous structures of the MTP1 and, therefore, induce a spontaneous correction of the IMA via a similar mechanism to that seen in MTP1 arthrodesis.

It is currently recognized that excessive pronation of the first metatarsal is common in HV [4,11,12,13] and could play a role in its pathogenesis [4,14,15]. Additionally, Conti et al. [16] showed that surgical correction of pronation in HV improves patient-reported outcomes and decreases the recurrence rate. This pronation is characterized by a rounded appearance of the head of the first metatarsal on weight-bearing dorsoplantar radiographs [17,18]. A valgus tilt of the distal articular surface of the first metatarsal is also present, although it is confounded by this rounded shape of the M1 head induced by its pronation [19,20]. In this study, we propose a distal osteotomy, named the 3D chevron, which combines supination and varization of the M1 head. The arbitrary correction of these two bone deformities may realign the soft tissues around the MTP1 and allow for a spontaneous reduction in the IMA even in the absence of lateral translation of the M1 head.

Thus, our study aimed to answer the two following questions: (1) Does the 3D chevron correct HV without lateral translation of the M1 head? (2) Are spontaneous reductions in IMAs present in distal M1 osteotomies, even in the absence of lateral translations of the M1 heads?

We hypothesized that spontaneous reductions in IMAs would be observed in distal M1 osteotomies without lateral translations of the heads.

## 2. Materials and Methods

### 2.1. Population

A prospective single-surgeon study was performed, including a consecutive series of 25 HV cases. Any patient with a painful and debilitating HV deformity, resistant to medical treatment, and for whom a surgical intervention was indicated met the inclusion criteria. Patients with HV with associated pathologies requiring procedures on the lateral metatarsals, significant nonreducible or arthritic HV warranting MTP1 arthrodesis, or recurrences of the deformity after surgical treatment were not included.

Enrollment began on 1 June 2020. From that date onward, 25 consecutive patients meeting the inclusion criteria underwent a distal M1 3D chevron osteotomy.

### 2.2. Surgical Procedures

All patients underwent surgery under locoregional anesthesia in a supine position, with a pneumatic tourniquet applied at the ankle.

**Figure 1:**
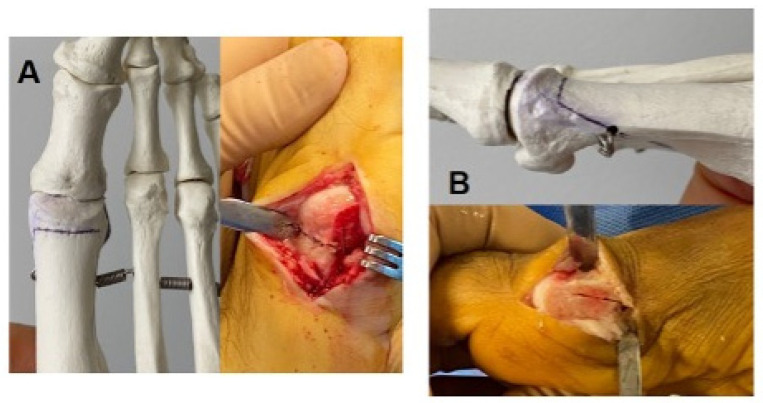
Representation of the cut lines of a standard Chevron osteotomy. (**A**): Dorsal view. (**B**): Medial view.

**Figure 2:**
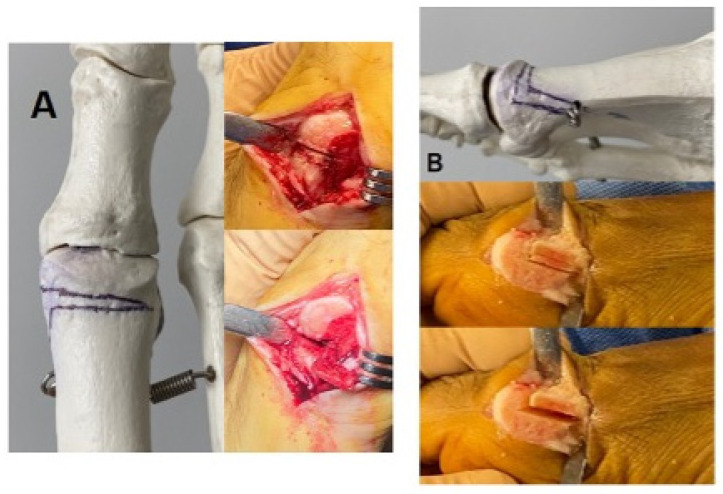
Representation of the cut lines of the 3D chevron osteotomy: (**A**) dorsal view; (**B**) medial view.

- Step 1: medial longitudinal incision centered on the MTP1, exposure of the joint capsule with protection of the medial dorsal sensory branch of the hallux, longitudinal capsulotomy centered on the MTP1, and exposure of the medial aspect of the head of the M1.
- Step 2: exostectomy.
- Step 3: Chevron osteotomy (Figure 1). The dorsal cut was vertical, made 3 mm proximal to the distal articular surface of the M1, orthogonal to the axis of the 2nd metatarsal and extended over the dorsal third of the M1 head. The plantar cut began at the end of the dorsal cut and ended at the level of the neck of the M1 with an angulation of approximately 120°. The orientation of the saw in the dorsoplantar plane was aligned with the 4th metatarsal.
- Step 4: Sectioning of the lateral metatarso-sesamoid ligament (i.e., suspensory ligament) through osteotomy. The phalangeal and lateral sesamoid insertions of the conjoined tendon of the adductor muscle were not severed.
- Step 5: in the plantar part of the osteotomy, a medial wedge resection extending across the entire width of M1, with a Akin osteotomy thickness of 3 mm at its base, was performed, allowing for a supination movement of the head (Figure 2).

In the dorsal part of the osteotomy, a second cut parallel to the distal articular surface of the M1, joining the first dorsal cut, was performed, allowing for medial wedge resection resulting in varization of the head (Figure 2).

Subsequently, the chevron was fixed in place, without translation, using a pin. A fluoroscopic radiographic evaluation was performed using a simulated load on the foot. If the IMA was less than 10°, HVA less than or equal to 15°, and the Hardy and Clapham score less than or equal to 4, the chevron was fixed using a screw, again without translation; these hallux valgus cases were then classified in the group labeled “successful correction without translation”. If these criteria were not met, a lateral translation of the head of the M1 head was carried out to complete the correction, and the chevron was then fixed using a screw. These hallux valgus cases were classified in the group labeled “failed correction without translation” (the amount of translation performed was adjusted until achieving the desired correction).

- Step 6: implementation of an Akin osteotomy fixed with a screw [21].
- Step 7: release of the tourniquet; hemostasis; closure without capsulorrhaphy.

### 2.3. Collecting Radiographic Data

Standardized weight-bearing dorsoplantar foot radiographs were taken and analyzed during the preoperative consultation [21]. The IMA, hallux valgus angle (HVA), distal metatarsal articular angle (DMAA), and Hardy and Clapham score [22], as well as the appearance of the lateral part of the head of the M1 according to the Okuda classification, were collected [18]. Okuda et al. [18] described the three following possible aspects of the lateral M1 head: angled (A), intermediate (I), or rounded (R). According to Ono et al. [4], the Okuda classification is correlated with the pronation of the first metatarsal, as follows: a rounded head (R) indicates an excessive pronation of the first metatarsal, and an angled head (A) indicates its absence.

A weight-bearing dorsoplantar foot X-ray was also conducted and analyzed using the same criteria during the one-year postoperative follow-up consultation.

In accordance with the literature and to reduce the variability in the measurements, the axis of the first metatarsal chosen for these measurements was the axis passing through the center of the articular surface of the first cuneo-metatarsal joint and through the center of the metatarsal head [23].

For some of these criteria, particularly DMAA and the Okuda classification, which did not demonstrate inter-observer reliability in the literature, a blind reading by two fellowship trained foot and ankle surgeons was carried out on both preoperative and postoperative X-rays.

### 2.4. Assessment Criteria

The criteria enabling us to address Question 1 were collected both preoperatively and intraoperatively. The surgical procedure was performed as described above. Two groups were formed during surgery. Patients who did not require lateral translation of the M1 head entered the “successful correction without translation” group, and patients who required translation entered the “failed correction without translation” group. The comparison of the preoperative radiographic data of these two groups addresses Question 1.

In order to address Question 2, the IMA measured on the preoperative radiograph was compared to the IMA measured on the radiograph at 1 year. This analysis was only performed in the “successful correction without translation” group.

### 2.5. Statistics

The interclass correlation coefficients (ICCs) were calculated in order to evaluate the inter-observer variability of radiographic analyses [24]. The normality of the variables was tested with the Shapiro–Wilk test. Non-normal variables were compared using a Mann–Whitney test and normal variables using a Student’s *t*-test. Comparison among groups according to the Okuda classification was performed using the Chi-square test. Significance was set at α < 5%. The statistical analysis was carried out with EasyMedStat (version 3.20.4; www.easymedstat.com, accessed on 1 June 2024).

## 3. Results

The entire patient cohort, including 22 women and 3 men, with a mean age of 44.8 ± 14.2 years and a mean body mass index of 22.6 ± 4.1 kg/m^2^ underwent follow up at one year after surgery.

Inter-observer reproducibility was excellent for IMA, HVA, Hardy and Clapham score, as well as for the Okuda classification. It was good for DMAA (=0.61).

In response to our Question 1, correction of the deformity without lateral translation of the head of M1 was possible for 13 out of 25 patients (52%). These individuals constituted the “successful correction without translation” group. The remaining 12 patients composed the “failed correction without translation” group. Preoperative comparisons between these two groups revealed significantly lower IMAs and HVA values in the “successful correction without translation” group (median IMA = 13 (IQR 2), median HVA = 25 (IQR 3) compared to the group “failed correction without translation” (median IMA = 16 (IQR 2.25) *p* < 0.001, median HVA = 35 (IQR 2.5)); *p* = 0.001). The DMAA was also significantly lower in the “successful correction without translation” group (*p* = 0.002), while no significant differences was observed for all other criteria (Table 1).

**Figure 3:**
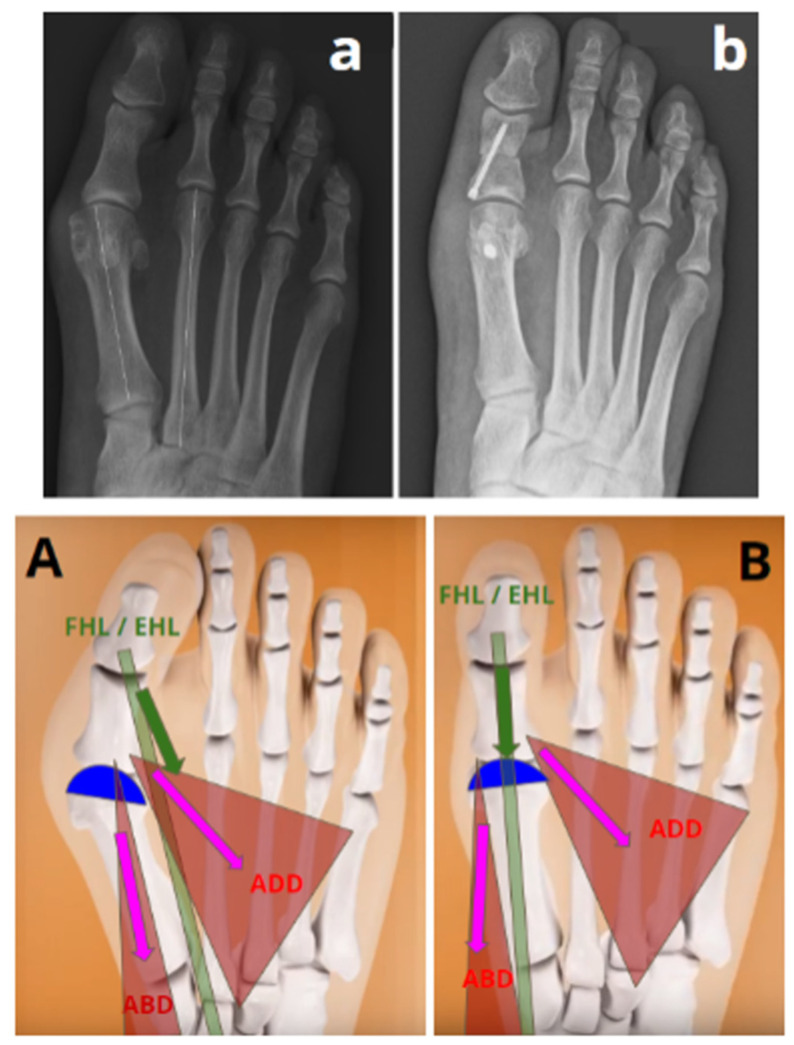
Representation of a spontaneous reduction in the IMA after 3D chevron osteotomy without lateral translation of the first metatarsal head (“successful without translation” group). ADD = adductor hallucis muscle; ABD = abductor hallucis muscle; FHL = tendon of the flexor hallucis longus muscle; EHL = tendon of the extensor hallucis longus muscle. (**A**) The deformity in the valgus and pronation of the distal epiphysis of the first metatarsal (represented in blue) induces an imbalance in the adjacent musculotendinous structures. (**a**) Preoperative radiography of a hallux valgus in the “successful correction without translation” group with an IMA at 13°. (**B**) An osteotomy combining supination and varization allows for a correction of this deformity, resulting in the balance of the adjacent musculotendinous structures, allowing for the spontaneous reduction in the IMA. (**b**) Postoperative radiography showing a reduction in the IMA at 6° without translation.

**Figure 4:**
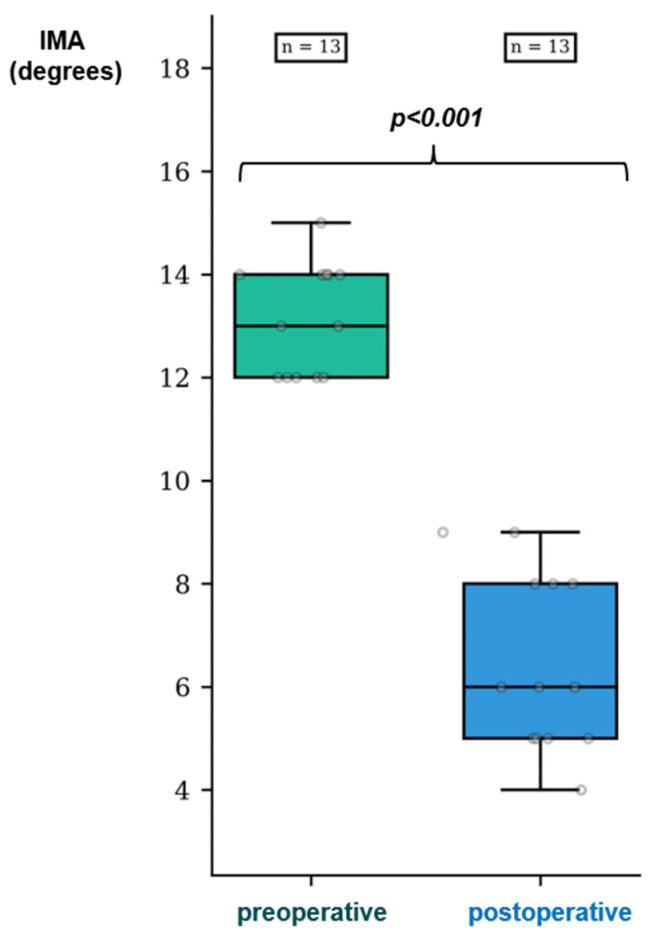
Pre- and postoperative distribution of the IMAs in the “successful correction without translation” group.

In response to Question 2, in the group “successful correction without translation”, spontaneous reduction in IMA in the absence of lateral translation of the head was observed with a median value of 13° (IQR 2) preoperatively versus 6° (IQR 3) at 1 year postoperatively, showing a median difference of 6° (IQR = 1; CI95% = [5.5; 8.0]; *p* < 0.001) (Figure 3 and Figure 4).

The five following complications were noted: two cases of pain at the metatarsal screw site (one in each group), including one requiring surgical revision for early removal (“failed correction without translation” group); two asymptomatic recurrences of the deformity (one in the “success” group (HVA = 17°), one in the “failed correction without translation” group (HVA = 18°)); and one onset of transfer metatarsalgia treated medically.

Angular results at 1 year for the “successful correction without translation” group are shown in Table 2 and in Table 3 for the “failed correction without translation” group.

## 4. Discussion

Our study aimed to assess whether spontaneous reduction in the IMA can be present in distal M1 osteotomies in the absence of lateral translation of the M1 head in HV surgical corrections. The 3D chevron, by combining supination and varization osteotomies of the M1 distal articular surface, was able to obtain HV correction without lateral translation of the M1 head but only in mild deformities. In these cases, even in the absence of an M1 head lateral displacement, there was a spontaneous correction of the IMA. Thus, our hypothesis was confirmed.

According to Cronin et al. [25], the spontaneous reduction in the IMA has to be ascribed to the action of the hallux adductor muscle, which, following arthrodesis of the first metatarsophalangeal joint (MTP1), functions as an adductor for the entire first ray rather than as an adductor of the hallux. We assumed a similar mechanism in our study. The lateral offsetting of the tendons of the flexor and extensor hallucis longus, as well as the plantarization and lateralization of the distal insertion of the abductor hallucis due to pronation, creates a valgus force on the hallux [26]. Supination and varus correction provided by this osteotomy enabled a rebalancing of the adjacent musculotendinous structures around the MTP1, facilitating a spontaneous IMA reduction under the influence of the adductor hallucis (Figure 3). This phenomenon may have also occurred in the “failed correction without translation” group of our study, as we performed supination and varization osteotomies in a similar manner to rebalance the soft tissues. We have decided to divide the results of our study between the “successful correction without translation” group and the “failed correction without translation” group, as in the latter we were unable to differentiate whether the IMA correction observed stems from this automatic phenomenon or from the lateral displacement of the M1 head through the osteotomy itself.

The 3D chevron presents some similarities with the Reverdin and Isham osteotomy [27], whereby a medial wedge resection without translation corrects the varus of the M1 distal epiphysis. While the 45° oblique nature of the osteotomy, relative to the M1 shaft, has been described to prevent dorsal displacement of the head and allow for immediate weight-bearing, it also leads to a supination correction like in the 3D chevron. However, the spontaneous reduction in the IMA was not completely observed in Reverdin and Isham osteotomies [28,29]. We believe that the absence of correction can be explained by the achievement of lateral release performed in these series, including tendon insertions of the adductor muscle. It is also possible that the oblique nature of the osteotomy might not be sufficient to correct the pronation of the M1 head.

We cannot confirm whether the 3D chevron is likely to improve our clinical and radiological outcomes, as this study was not designed to address these questions. However, we noted a decrease in the rounded appearance of the head postoperatively, which could be secondary to the supination performed in our osteotomy (Table 2) [30]. The observed DMAA reduction could result from both varus correction and supination. The measurement of the valgus tilt of the distal articular surface of M1 by this angle is overestimated because of the roundness of the head secondary to the pronation of the M1 [20]. Both criteria are recognized as risk factors for the recurrence of deformity in HV in the event of nonreduction postoperatively [18,31], making their improvement through the 3D chevron noteworthy. Comparative studies are needed for further clarification.

Our study has several limitations. The small sample size in our series prevents a reliable evaluation of the outcomes of this technique. However, our objective was to evaluate the phenomenon of spontaneous reduction in the IMA in distal osteotomies without lateral translation of the M1 head. A significant result was observed in this aspect of our study, demonstrating sufficient statistical power to address this question. The inter-observer reproducibility of DMAA (ICC = 0.61) was only 0.02 points from being poor (ICC < 0.6). Therefore, our findings on this measure should be interpreted cautiously. This angle is often overlooked in the literature, as pronation tends to overestimate the tilt toward valgus of the distal articular surface of the M1 [20]. We did not evaluate MTP1 incongruence in our study, which could have been a factor influencing the study’s results. Nevertheless, MTP1 incongruence is a challenging factor to assess on dorsoplantar radiographs, as is the DMAA, and the additional inclusion of this parameter may have led to erroneous conclusions. Further studies including weight-bearing CT images are needed to properly assess MTP1 incongruence. We did not study the intra-observer reproducibility of the different radiological parameters in our study; however, these are commonly used measurements that have already demonstrated good intra-observer reproducibility in the literature [18,21]. Additionally, preoperative radiographs were taken with simulated weight-bearing, which could introduce biases to angular values and group composition. However, the aim of our study was not to compare these groups postoperatively but rather to investigate the reduction in the IMA when no lateral translation of the M1 head was performed. This variable was assessed using weight-bearing preoperative and 1-year postoperative radiographs, exclusively in the “successful correction without translation” group. The reliability of Okuda’s classification for indirectly assessing metatarsal pronation has been criticized in two recent studies [32,33]. Our results on this secondary endpoint should, therefore, be interpreted with caution. Direct measurements of the pronation on sesamoid incidences could have limited this bias but are difficult to carry out in clinical practice [12]. The exclusion of HV cases presenting with metatarsalgia requiring lateral osteotomies indirectly excluded patients with the most severe deformities from our study. It is likely that the inclusion of these patients would have increased the number of feet for which a 3D chevron without lateral translation of the M1 head was impossible (i.e., the group labeled “failed correction without translation”). However, this limit has no impact on the results of the “successful correction without translation” group, which included HVs with the most minor deformities, and the evaluation of spontaneous correction of the IMA was only judged for this group. Furthermore, we did not quantify the likely presence of flat feet combined with HV in our series. This factor is reported to influence certain HV parameters, particularly in the coronal plane, but does not influence the radiographic measurement in the dorsoplantar plane [4,34]. No correction of flat feet was performed during surgery, and, thus, the comparison of the same feet in pre- and postoperative assessments was not affected by this potential bias. In addition, the HV pathogenesis is likely to be multifactorial, and this study assessed only M1 dysplasia without taking into account other potential risk factors present in the literature [35,36]. Finally, we did not collect clinical parameters in our study; therefore, we cannot assert that the spontaneous reductions in the IMAs in distal osteotomies without lateral translations of the M1 heads are accompanied by an improvement in patients’ symptoms.

This study provided an interesting pathophysiological illustration of how the interaction between bone and soft tissue across the first ray is crucial in the HV pathogenesis and surgical correction. Currently, high rates of recurrence are observed, particularly in long-term follow-up [5]. It is likely that realigning the bone without recreating a perfect balance with the surrounding soft tissue may lead to slow recurrence over time. Despite the limitations mentioned above, the results of our study highlighted the importance of recreating the balance between bone and soft tissue in HV in a comprehensive manner to improve surgical outcomes.

## 5. Conclusions

Despite the absence of the lateral translation of the M1 head, 3D chevron osteotomy allowed for spontaneous reductions in the IMAs in HV cases with mild deformities. Therefore, lateral translation of the M1 head should not be considered the only method for correcting IMAs in distal osteotomies. Correcting HV without laterally displacing the M1 head might result in a better balance between first ray bony structures and soft tissues. With this in mind, future studies should assess whether lateral displacement of the M1 head to correct HV is a potential destabilizer of the first ray, especially the first tarsometatarsal joint.

## Figures and Tables

**Table 1:** Comparison of preoperative radiographic data from the “successful correction without translation” and “failed correction without translation” groups.

	IMA (Degrees) Median (IQR)	HVA (Degrees) Median (IQR)	DMAA (Degrees) Mean ± SD	Hardy and Clapham Score Median (IQR)	Okuda Classification
Successful Group (n= 13)	13 (IQR 2)	25 (IQR 3)	14.5 ± 5.5	6 (IQR 2)	2 A/3 I/8 R
Failed Group % (n = 12)	16 (IQR 2.25)	35 (IQR 2.5)	20.9 ± 6.9	6 (IQR 1.25)	0 A/5 I/7 R
***p-Value***	***0.0006***	***0.001***	***0.02***	*0.31*	*0.28*

IQR = interquartile range; SD = standard deviation; A = angulated; I = intermediate; R = rounded; IMA = intermetatarsal angle; HVA = hallux valgus angle; DMAA = distal metatarsal articular angle.

**Table 2:** Angular results at 1 year for the “successful correction without translation” group.

	IMA (Degrees) Median (IQR)	HVA (Degrees) Mean ± SD	DMAA (Degrees) Mean ± SD	Hardy and Clapham Score Median (IQR)	Okuda Classification
**Preoperative** (n = 13)	13 (IQR 2)	25.4 ± 3.1	14.5 ± 5.5	6 (IQR 2)	2A/3I/8R
**1 year** (n = 13)	6 (IQR 3)	11.5 ± 4.0	4.8 ± 2.9	3 (IQR 2)	7A/6I/0R
***p-Value***	***<0.001***	***<0.001***	***<0.001***	***0.002***	***<0.001***

IQR = interquartile range; SD = standard deviation; A = angulated; I = intermediate; R = rounded; IMA = intermetatarsal angle; HVA = hallux valgus angle; DMAA = distal metatarsal articular angle.

**Table 3:** Angular results at 1 year for the “failed correction without translation” group.

	IMA (Degrees) Mean ± SD	HVA (Degrees) Median (IQR)	DMAA (Degrees) Mean ± SD	Hardy and Clapham Score Median (IQR)	Okuda Classification
**Preoperative** (n = 12)	16.3 ± 2.4	35 (IQR 2.5)	20.9 ± 6.9	6 (IQR 1.25)	0A/5I/7R
**1 year** (n = 12)	5.7 ± 2.1	12 (IQR 7.8)	7.3 ± 3	2 (IQR 1.25)	5A/6I/1R
***p-Value***	***<0.001***	***<0.001***	***<0.001***	***<0.001***	***<0.001***

IQR = interquartile range; SD = standard deviation; A = angulated; I = intermediate; R = rounded; IMA = intermetatarsal angle; HVA = hallux valgus angle; DMAA = distal metatarsal articular angle.

## Data Availability

The datasets generated and/or analyzed during the current study are available from the corresponding author on reasonable request due to privacy.

## References

[1] Barg A., Harmer J.R., Presson A.P., Zhang C., Lackey M., Saltzman C.L. (2018). Unfavorable Outcomes Following Surgical Treatment of Hallux Valgus Deformity: A Systematic Literature Review. J. Bone Jt. Surg..

[2] Santos M., Roseiro L., Seiça E.C., Amaro A.M. (2024). A Systematic Review of Osteotomies to Correct Hallux Valgus in the First Metatarsal. Appl. Sci..

[3] Jeuken R.M., Schotanus M.G.M., Kort N.P., Deenik A., Jong B., Hendrickx R.P.M. (2016). Long-Term Follow-up of a Randomized Controlled Trial Comparing Scarf to Chevron Osteotomy in Hallux Valgus Correction. Foot Ankle Int..

[4] Lalevée M., Barbachan Mansur N.S., Dibbern K., Briggs H., Maly C.J., de Carvalho K.A.M., Lintz F., de Cesar Netto C. (2022). Coronal Plane Rotation of the Medial Column in Hallux Valgus: A Retrospective Case-Control Study. Foot Ankle Int..

[5] Lalevee M., de Cesar Netto C., ReSurg, Boublil D., Coillard J.Y. (2023). Recurrence Rates with Longer-Term Follow-up After Hallux Valgus Surgical Treatment with Distal Metatarsal Osteotomies: A Systematic Review and Meta-Analysis. Foot Ankle Int..

[6] Steadman J., Bakshi N., Philippi M., Arena C., Leake R., Barg A., Saltzman C.L. (2022). Association of Normal vs Abnormal Meary Angle with Hindfoot Malalignment and First Metatarsal Rotation: A Short Report. Foot Ankle Int..

[7] Murawski D.E., Beskin J.L. (2008). Increased Displacement Maximizes the Utility of the Distal Chevron Osteotomy for Hallux Valgus Deformity Correction. Foot Ankle Int..

[8] Vernois J., Redfern D.J. (2016). Percutaneous Surgery for Severe Hallux Valgus. Foot Ankle Clin..

[9] Dalat F., Cottalorda F., Fessy M.H., Besse J.L. (2015). Does Arthrodesis of the First Metatarsophalangeal Joint Correct the Intermetatarsal M1M2 Angle? Analysis of a Continuous Series of 208 Arthrodeses Fixed with Plates. Orthop. Traumatol. Surg. Res..

[10] Dayton P., Carvalho S., Egdorf R., Dayton M. (2020). Comparison of Radiographic Measurements Before and After Triplane Tarsometatarsal Arthrodesis for Hallux Valgus. J. Foot Ankle Surg..

[11] Kim Y., Kim J.S., Young K.W., Naraghi R., Cho H.K., Lee S.Y. (2015). A New Measure of Tibial Sesamoid Position in Hallux Valgus in Relation to the Coronal Rotation of the First Metatarsal in CT Scans. Foot Ankle Int..

[12] Mortier J.P., Bernard J.L., Maestro M. (2012). Axial Rotation of the First Metatarsal Head in a Normal Population and Hallux Valgus Patients. Orthop. Traumatol. Surg. Res..

[13] Najefi A.A., Malhotra K., Patel S., Cullen N., Welck M. (2022). Assessing the Rotation of the First Metatarsal on Computed Tomography Scans: A Systematic Literature Review. Foot Ankle Int..

[14] Najefi A.A., Katmeh R., Zaveri A.K., Alsafi M.K., Garrick F., Malhotra K., Patel S., Cullen N., Welck M. (2022). Imaging Findings and First Metatarsal Rotation in Hallux Valgus. Foot Ankle Int..

[15] Steadman J., Barg A., Saltzman C.L. (2021). First Metatarsal Rotation in Hallux Valgus Deformity. Foot Ankle Int..

[16] Conti M.S., Patel T.J., Zhu J., Elliott A.J., Conti S.F., Ellis S.J. (2022). Association of First Metatarsal Pronation Correction with Patient-Reported Outcomes and Recurrence Rates in Hallux Valgus. Foot Ankle Int..

[17] Ono Y., Yamaguchi S., Sadamasu A., Kimura S., Watanabe S., Akagi R., Sasho T., Ohtori S. (2020). The Shape of the First Metatarsal Head and Its Association with the Presence of Sesamoid-Metatarsal Joint Osteoarthritis and the Pronation Angle. J. Orthop. Sci..

[18] Okuda R., Kinoshita M., Yasuda T., Jotoku T., Kitano N., Shima H. (2007). The Shape of the Lateral Edge of the First Metatarsal Head as a Risk Factor for Recurrence of Hallux Valgus. J. Bone Jt. Surg. Am..

[19] Jastifer J.R., Coughlin M.J., Schutt S., Hirose C., Kennedy M., Grebing B., Smith B., Cooper T., Golano P., Viladot R. (2014). Comparison of Radiographic and Anatomic Distal Metatarsal Articular Angle in Cadaver Feet. Foot Ankle Int..

[20] Lalevée M., Barbachan Mansur N.S., Lee H.Y., Maly C.J., Iehl C.J., Nery C., Lintz F., de Cesar Netto C. (2022). Distal Metatarsal Articular Angle in Hallux Valgus Deformity. Fact or Fiction? A 3-Dimensional Weightbearing CT Assessment. Foot Ankle Int..

[21] Lee K.M., Ahn S., Chung C.Y., Sung K.H., Park M.S. (2012). Reliability and Relationship of Radiographic Measurements in Hallux Valgus. Clin. Orthop. Relat. Res..

[22] Hardy R.H., Clapham J.C. (1951). Observations on Hallux Valgus; Based on a Controlled Series. J. Bone Jt. Surg. Br..

[23] Shima H., Okuda R., Yasuda T., Jotoku T., Kitano N., Kinoshita M. (2009). Radiographic Measurements in Patients with Hallux Valgus before and after Proximal Crescentic Osteotomy. J. Bone Jt. Surg. Am..

[24] Shrout P.E., Fleiss J.L. (1979). Intraclass Correlations: Uses in Assessing Rater Reliability. Psychol. Bull..

[25] Cronin J.J., Limbers J.P., Kutty S., Stephens M.M. (2006). Intermetatarsal Angle after First Metatarsophalangeal Joint Arthrodesis for Hallux Valgus. Foot Ankle Int..

[26] Welck M.J., Al-Khudairi N. (2018). Imaging of Hallux Valgus: How to Approach the Deformity. Foot Ankle Clin..

[27] Sa I. (1991). The Reverdin-Isham Procedure for the Correction of Hallux Abducto Valgus: A Distal Metatarsal Osteotomy Procedure. Clin. Podiatr. Med. Surg..

[28] Bauer T., Biau D., Lortat-Jacob A., Hardy P. (2010). Percutaneous Hallux Valgus Correction Using the Reverdin-Isham Osteotomy. Orthop. Traumatol. Surg. Res..

[29] Severyns M., Carret P., Brunier-Agot L., Debandt M., Odri G.A., Rouvillain J.L. (2019). Reverdin-Isham Procedure for Mild or Moderate Hallux Valgus: Clinical and Radiographic Outcomes. Musculoskelet. Surg..

[30] Yasuda T., Okuda R., Jotoku T., Shima H., Hida T., Neo M. (2015). Proximal Supination Osteotomy of the First Metatarsal for Hallux Valgus. Foot Ankle Int..

[31] Park C.H., Lee W.C. (2017). Recurrence of Hallux Valgus Can Be Predicted from Immediate Postoperative Non-Weight-Bearing Radiographs. J. Bone Jt. Surg. Am..

[32] Mansur N.S.B., Lalevee M., Schmidt E., Dibbern K., Wagner P., Wagner E., de Souza Nery C.A., de Cesar Netto C. (2021). Correlation between Indirect Radiographic Parameters of First Metatarsal Rotation in Hallux Valgus and Values on Weight-Bearing Computed Tomography. Int. Orthop..

[33] Patel T.J., Conti M.S., Caolo K.C., Miller M.C., Conti S.F., Ellis S.J. (2022). Pronation on Weightbearing Radiographs Does Not Correlate with Pronation from Weightbearing CT Scans. Foot Ankle Surg..

[34] Tsikopoulos K., Papaioannou P., Kitridis D., Mavridis D., Georgiannos D. (2018). Proximal versus Distal Metatarsal Osteotomies for Moderate to Severe Hallux Valgus Deformity: A Systematic Review and Meta-Analysis of Clinical and Radiological Outcomes. Int. Orthop..

[35] Zhu C., Song Y., Xu Y., Zhu A., Baker J.S., Liu W., Gu Y. (2024). Toe Box Shape of Running Shoes Affects In-Shoe Foot Displacement and Deformation: A Randomized Crossover Study. Bioengineering.

[36] Perera A.M., Mason L., Stephens M.M. (2011). The pathogenesis of hallux valgus. J. Bone Jt. Surg. Am..

